# The Cobra Catheter: A Novel Concept and Device for the Thru-Septal Myocardium Approach

**DOI:** 10.1155/2022/7500175

**Published:** 2022-05-06

**Authors:** Min-Ku Chon, Dong-Hoon Shin, Su-Jin Jung, June-Hong Kim

**Affiliations:** ^1^Department of Cardiology, School of Medicine, Pusan National University and Cardiovascular Center, Yangsan Hospital, Yangsan (50612), Republic of Korea; ^2^Department of Pathology, School of Medicine, Pusan National University, Yangsan Hospital, Yangsan (50612), Republic of Korea; ^3^Tau-PNU MEDICAL Co.Ltd., Department of Research Planning, R&D Center, Yangsan (50612), Republic of Korea

## Abstract

**Objectives:**

In our previous study, we suggested the novel septal traversing technique as effective and safe in catheter-based approach for septal myocardium. However, it is limited by its dependence on the septal perforator vein. This study aimed to evaluate the Cobra catheter as a backup catheter to overcome this limitation in swine.

**Methods:**

We designed the guiding Cobra catheter. It consisted of three major parts (the external pull-wire steerable distal tip, the C-shaped shaft, and the steering adjustment handle). We tested the difference in force between the guidewire passing through the muscle and the vessel wall using a push-pull gauge. We performed a septal wire engage procedure in swine using the Cobra catheter. The guidewire engagement of the septal vein and Cobra catheter were compared visually and histopathologically.

**Results:**

A total of ten swine were enrolled in this study. The success rate was 100% under fluoroscopy. The experiments confirmed the medical potential of the septal approach even in a location irrelevant to the septal perforator vein anatomy and confirmed that the wire passed well in the target direction in the harvested heart. There was no serious physical damage or pathological abnormalities in the vessel wall and myocardium.

**Conclusion:**

These results showed that the novel Cobra catheter with a septal vein-independent trans-septal approach may be a safe and effective alternative for the treatment of structural heart diseases.

## 1. Introduction

The cardiovascular community has recently been interested in catheter-based minimally invasive treatments as an alternative to conventional heart surgery [1,2]. These catheter-based minimally invasive treatments may be in the form of a novel medical device, technology, or technique [1,2]. In our previous study, we had proposed a new treatment methodology (called Thru-Septal technology) in which the myocardial septum is approached through the coronary sinus. This approach has been used in the development of treatment techniques for mitral valve regurgitation through mitral loop cerclage, physiologic pacing (previously called cerclage pacing), and septal reduction by intraseptal radiofrequency (RF) ablation (previously called cerclage RF ablation) for hypertrophic cardiomyopathy (HOCM). A preclinical study showed notable results of the Thru-Septal approach in treating mitral regurgitation. In the first trial of this technique in humans, its therapeutic feasibility, efficacy, and safety as a novel approach for catheter-based mitral valve regurgitation treatment were confirmed [3–5].

The physiological septal pacing technology through the coronary sinus and septal vein is a novel concept of the Para-Hisian pacing that offers an alternative approach to His pacing by taking anatomical advantage of the septal vein in approaching the His bundle. The natural curvature of the pacing lead contributes to the achievement of a more stable lead positioning and maintenance and a narrow QRS pace. These features make the Thru-Septal pacing an alternative to direct right-sided approaches to His bundle pacing. In addition, some studies that attempt to approach the ventricular septum through the septal perforator vein are introduced [6,7]. A study had examined the trans-coronary intraseptal RF ablation technique in the treatment of HOCM [8], which had shown that the utilization of the Thru-Septal RF ablation was eligible for evaluation in a clinical trial. However, Thru-Septal technologies are dependent on the septal perforator vein anatomy in approaching the basal interventricular septum (IVS) from the distal part of the coronary sinus. This dependence is a major limitation of this approach. To overcome this limitation, this study aimed to evaluate a new device—the Cobra catheter—in enabling the guidewire to traverse or engage the septal myocardium without using the septal perforator vein.

## 2. Materials and Methods

### 2.1. Device

**Figure 1:**
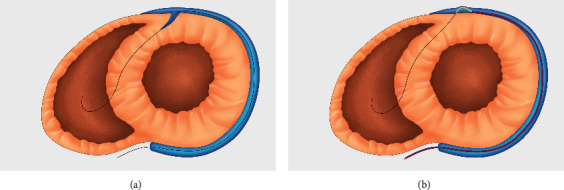
The conceptual illustration of (a) the guidewire engaging the myocardium of patients through the septal vein and (b) the role of the Cobra catheter in patients without a septal vein.

**Figure 2:**
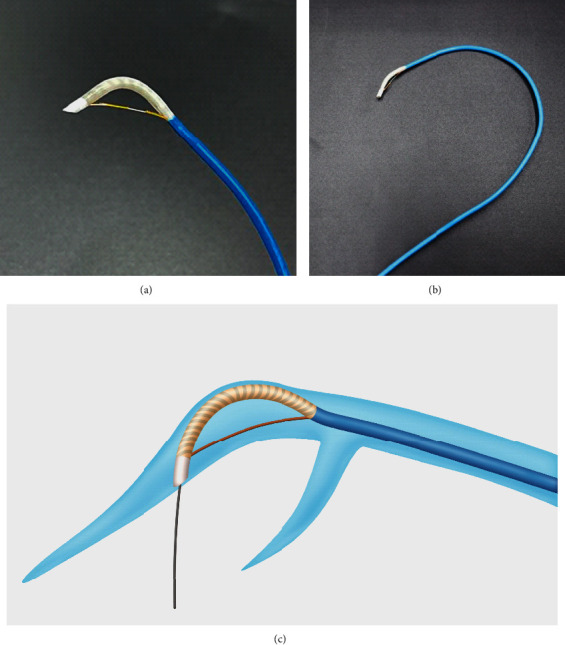
The Cobra catheter design. (a) The distal tip of the Cobra catheter is oblique-shaped to establish complete contact with the vessel wall. The wire outside the sheath creates a bending moment when the wire is pulled by the rotating handle. (b) The preshaped catheter shaft provides an appropriate degree of angulation and torquability to guide the guidewire towards the desired direction. The curve matches the prospective shape of the coronary sinus. (c) This design provides an adequate backup force to directly engage the guidewire through the punctured vessel structure.

The Cobra catheter was fabricated by Tau-PNU Medical (Busan, South Korea) according to the investigator's proposal (Figure 1). It had three major parts: the steerable distal tip, the C-shaped body shaft, and the steering adjustment handle. The outer diameter of the distal tip was 1.75 mm (5.25 Fr), while the length of the steerable section was 15 mm. The tip had a marker band that connected the pull-wire. This was the steerable section of the catheter. The wire outside the sheath created a bending motion when the wire was pulled by the rotating handle. The distal tip of the Cobra catheter was constructed to be oblique to establish complete contact with the vessel wall in order to provide stability while puncturing the direct vessel structure. The catheter shaft was steered tight to the vessel wall in the opposite direction from the tip (Figure 2(a) and 2(c)). This design provided an adequate backup force in engaging the guidewire to directly puncture the vessel structure. The distal shaft of the Cobra catheter resembled a C-shape (Figure 2(b)) to match the prospective shape of the coronary sinus. This structure provided an appropriate degree of angulation and torquability in guiding the catheter towards the desired direction. Furthermore, the rotation of the handle allowed the bending of the tip by pulling the pull-wire.

### 2.2. Guidewire Engage Function Test

The septal vein provided a natural path for entering the myocardium without massive resistance. It also enabled access to the myocardium through a direct puncture with a guidewire. For the guidewire to engage through the vessel wall to gain sufficient force to pass, a backup force was required. Thus, we conducted a guidewire engage function test to examine a new catheter guide that could provide sufficient backup force to directly puncture the vascular structure and access the myocardium with a guidewire.

First, we prepared a push-pull gauge (Optech, DS2-20N model) that measured the force needed to push the wire. The push-pull gauge was connected to the experimental 0.014' guidewire (ASHAHI Intec, Japan) and to the distal end of the push-pull gauge. In the harvested heart, the end of the guidewire was pushed into the myocardium. The force required for the guidewire to pass through the myocardium was measured. In the same way, the force required to engage the myocardium by directly puncturing the vessel structure was also measured.

### 2.3. Procedure

In the first step of the procedure, we placed an optimal sized-sheath in the jugular, subclavian, or the femoral vein and engaged the coronary sinus with a 0.035′ guidewire. The balloon-tipped guiding catheter (8 Fr, Cello, Medtronic) was engaged into the coronary sinus to perform a pressurized venogram, which confirmed the presence of the septal vein.

A 0.014′ guidewire and a Cobra catheter were inserted into the great cardiac vein. From there, the proper area and direction to engage the septal myocardium were found. At this point, the distal tip of the Cobra catheter was bent into an appropriate position and orientation. Its shape was maintained to provide sufficient backup force. This guide catheter was newly proposed as a procedural accessory device that allowed a safe and easy introduction of the guidewire into the septal myocardium in cases that lacked a proper septal vein. The guidewire was then changed to a 0.014-inch stiff wire to directly puncture the vascular structure and engage into the myocardium to traverse the septum.

Once the guidewire entered the myocardium, it was moved towards the desired direction and location. If the guidewire was directed to an inappropriate position, such as towards the pericardial space, it was verified through X-ray and echocardiogram (Transthoracic Echocardiography, TTE) and repositioned. The torque and angle of the catheter were manipulated to readjust it to the correct direction.

### 2.4. Estimation

During the procedure, echocardiography was used to confirm the guidewire's position inside the septum within the beating heart. After euthanasia, the position of the guidewire and myocardial damage were checked visually by opening the chest and harvesting the heart. Experienced specialists conducted pathological examinations, the results of which were concluded in a consensus. The results were based on comparison and analysis of vascular and myocardial damage. These structures were particularly examined because the guidewire engaged and directly punctured them with the Cobra catheter to reach the septal vein.

### 2.5. Animals

All animals were handled in accordance with the guidelines of the National Institute of Health and the American Physiological Society, as well as the policies of the Animal Care and Use Committee of the Pusan National University Yangsan Hospital (PNUYH, Korea). All animals received humane care. All experimental protocols and studies were approved by the Institutional Review Board (IRB) of the PNUYH (IRB No.: 2021-021-A2C0). This study involved ten healthy Yorkshire farm swine. The animals were placed under general anesthesia with an anesthesia ventilator (Multiplus, EVD Type/Royal Medical, Korea). The state of anesthesia, heart rate, heart rhythm, respiratory rate, temperature, blood pressure, oxygen saturation, end tidal carbon dioxide, and electrocardiography were continuously monitored during the procedure. All procedures were carried out by a trained veterinarian. Intramuscular alfaxalone 5 mg/kg and intramuscular xylazine 2 mg/kg were administered, followed by isoflurane 3% for maintenance anesthesia. Before the endotracheal intubation, atropine sulfate 1 mg was administered intravenously. Tramadol hydrochloride 50 mg and gentamicin sulfate 80 mg were administered after intubation. The anesthetized animals were placed in the supine position, which was maintained by ensuring their legs were tied to the table with a fabric band. Heparin sodium 5000 U was administered intravenously after venous puncture. Vecuronium (a muscle relaxant) (0.1 mg/kg) was administered hourly. In the animals in which modeling was completed and follow-up monitored, euthanasia was achieved by intravenous administration of potassium chloride (3 g/20 ml) during anesthesia.

## 3. Results

### 3.1. Function

**Figure 3:**
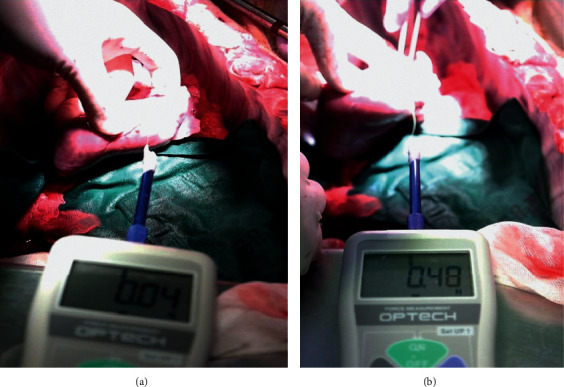
Procedural steps and wire positioning results. (a) The guidewire naturally passing through the myocardium with a force of 0.04 N (4.08 gf). (b) The guidewire requiring a force of 0.45 N (45.89 gf) to traverse the vessel wall and penetrate the myocardium.

The Cobra catheter aimed to provide backup force and support to the guidewire by directly puncturing the vascular structure and engaging the myocardium; for this, its angle, torque, and deflection needed to be easily controlled. To estimate the force required to pass through the tissue using a 0.014′ guidewire, the results were measured three times in the swine hearts. Experiments with push-pull gauges showed that the guidewire naturally passed through the myocardium with a force of 0.04 N (4.08 gf). On the contrary, 0.45 N (45.89 gf) was required to traverse the vasculature wall to allow the guidewire to penetrate through the myocardium (Figure 3). In other words, it required approximately 11 times more force to traverse the vessel wall through penetration with a guidewire as compared to traversing it naturally.

The procedures performed in the beating hearts of the swine were guided by X-ray fluoroscopic imaging. The device was visible on fluoroscopy, which made it convenient. Consequently, the guidewire passed through the ventricular septum without any functional abnormalities and was directed to the desired position within the ventricular septum muscle by the Cobra catheter.

The functions of the Cobra catheter such as its angle, torque, and deflection were all functional as we intended them to be. During the procedure, device-related side effects were not observed. Abnormalities and device failure due to the Cobra catheter were not observed in any of the procedures.

**Figure 4:**
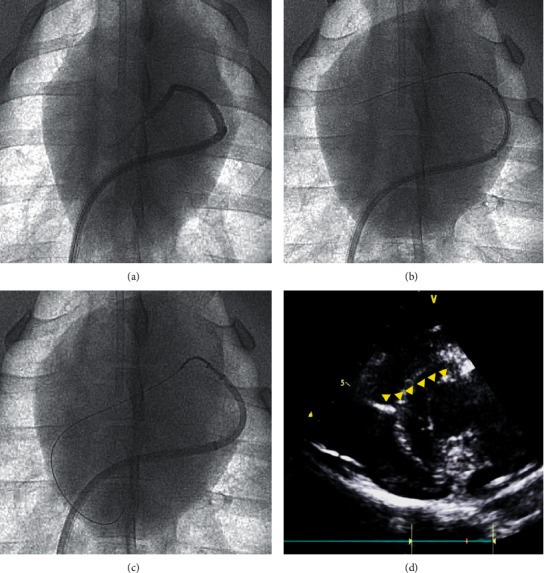
Procedural steps and wire positioning results. (a) Fluoroscopy confirms the anatomy without the septal vein. (b) The guidewire engages the Cobra catheter. (c) The Cobra catheter distal tip angulation towards the direction of the guidewire. (d) The guidewire in the septum.

During the beating-heart procedure (Figure 4), we confirmed that the guidewire was engaged through the septal myocardium with and without the septal vein.

After the beating-heart procedure, the heart was harvested to visually confirm the exit and path of the guidewire. The guidewires were successfully engaged into the septal myocardium. It was also confirmed that the guidewire was navigated to the desired location, just where the septal vein was present, even if it directly punctured the myocardium by the Cobra catheter without going through the septal vein.

**Figure 5:**
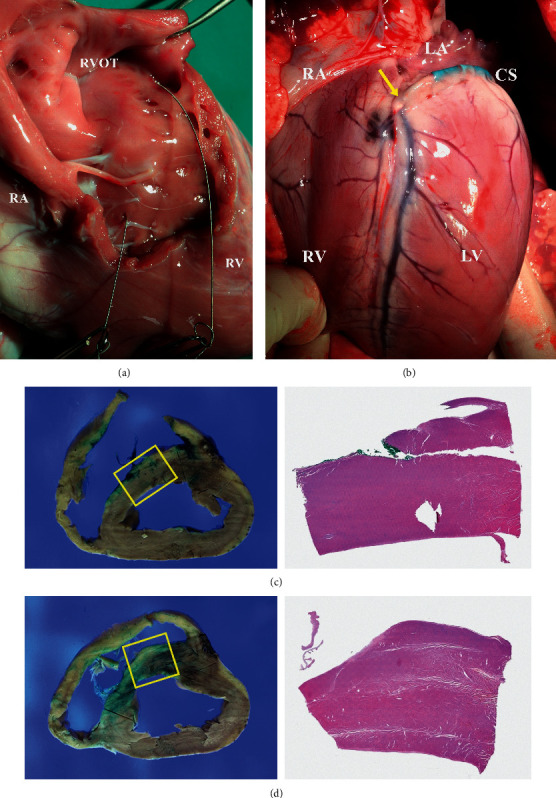
Visual inspection and pathology. Successfully engagement of the guidewire into the septal myocardium in both (a) the Thru-Septal approach and (b) by a direct myocardial puncture with the Cobra catheter. (c, d) Visible hemorrhagic lesion and foci with no ischemic change or necrosis. Slight hemorrhage without muscle damage in the posterior portions of the ventricle.

The pathology results are shown in Figure 5. Pathological analysis by experienced professionals showed no significant systematic damage of blood vessels and myocardial walls. There was no hemorrhage in the epicardium when the guidewire entered the myocardium through the septal vein. When visually checked in the harvested heart, a topical hematoma was observed around the guidewire puncture, but there was no spread of the hematoma or severe damage to other areas.

The formalin-fixed swine heart was sliced serially at 1.5 cm intervals from the apex to the valves. Sections from the upper portions of the IVS to the anterolateral portions of the left ventricle were submitted. Wired regions were marked with a green color.

Under microscopic examination, myocardial damage was not evident. In the myocardium, the hemorrhagic spots showed extravasated red blood cells and congestion. Some lesions showed hemorrhagic foci located on the anterolateral aspect of the left ventricle; these were identified as hemorrhage in the epicardium. The myocardium had hemorrhagic foci but did not contain any ischemic change or necrosis. The posterior portions of the ventricle showed hemorrhage without muscle damage. Although the guidewire engaged the septum using the Cobra catheter, the vessel wall and myocardial cells did not show evidence of any serious damage or abnormalities.

## 4. Discussion

This study showed that the novel design of the reentry Cobra catheter was very helpful in engaging the guidewire into the target IVS from the distal part of the coronary sinus without using the suitable septal perforator vein anatomy. Our study group has reported a couple of novel therapeutic technologies through the coronary sinus (called the cerclage family), such as the mitral loop cerclage, cerclage Para-Hisian pacing, and cerclage RF ablation. However, “cerclage” may not have been the appropriate terminology because pacing and RF ablation has nothing to do with circumferential tension, the origin of the cerclage.

As shown in our study, while penetrating the septal myocardium from the lumen of the coronary sinus, a significant pushing force, like piercing with a fine needle, is required because the outer wall of the coronary sinus is wrapped by a strong tissue. In contrast, the septal perforator vein has virtually no outer wall, which allows for a relatively easy control of wire navigation within the myocardium [6]. The Cobra catheter has an outer pulling wire, unlike the conventional deflectable catheter that has an internal pulling wire. This unique design strengthens the deflectable part of the catheter such that the guidewire can pierce the coronary sinus vessel wall into the targeted myocardium.

Another feature of the Cobra catheter is the preshape in its distal part. This preshape was designed to conform the coronary sinus anatomy into a C-shape. This shaping provided the deflectable part of the Cobra catheter a right orientation automatically. Otherwise, we would have to control the pointing direction of the deflection under the two-dimensional projection of fluoroscopic imaging; this is not only a frustrating and complex procedure but also a dangerous one because the wrong orientation of the deflectable tip may lead to perforation of the coronary sinus. However, with the preshape design, we were sure that the tip was always pointing downward when the deflection control handle was on.

The Cobra catheter also helped us control the direction of the guidewire while it traversed the IVS. In the mitral loop cerclage, the ideal wire exit into the right ventricle cavity was the right ventricular outflow tract area, whereas in the Thru-Septal pacing or in the RF ablation, a lower septum (medial papillary muscle of tricuspid valve) was preferred as the ideal wire exit site. This direction was indirectly controlled by the deflection angle of the Cobra catheter. The more the catheter was deflected, the lower the exit site of the wire was. This was easily controlled.

In this study, we proposed a novel device—the Cobra catheter, which has a special design of both the outer pull-wire and preshaping catheters—that may improve the existing vein-dependency limitation of septal perforators of the cerclage family of procedures. Cobra catheters may be applied in other procedures that require a strong backup support in directing a guidewire and may be an alternative to the conventional deflectable catheters.

This study has several limitations in terms of the anatomical structure of the septal vein. In order to evaluate the effectiveness of the Cobra catheter, a preclinical experiment was conducted on the heart of swine, which has the most similar anatomical structure to that of humans. However, swine heart have a slightly different direction and angle with humans; hence, there are some limitations in echocardiography. Unlike humans, swine have azygos veins immediately after entering the coronary sinus. During the procedure, this structure was the limitation when engaging the coronary sinus.

In addition, when using the Cobra catheter to cross the ventricular septum, we used a very fine wire (0.014 inch in diameter). The pathological examination confirmed that there were no tissue abnormalities, but there are still questions about the long-term effects of fibrotic scars, such as arrhythmia. This remains to be verified through further clinical trials.

## 5. Conclusion

In this study, a novel guiding catheter device—the Cobra catheter—was useful in the Thru-Septal approach through the venous system that did not rely on the septal vein. Therefore, we believe that this Cobra catheter technology may provide a medically effective treatment for structural heart disease that employs the septal vein-independent trans-septal approach.

## Data Availability

The image data including angiography, echocardiography, and pathology data used to support the findings of this study are included within the article.
